# Autocatalysis, Autopoiesis, and the Opportunity Cost of Individuality

**DOI:** 10.3390/biomimetics9060328

**Published:** 2024-05-30

**Authors:** Nemanja Kliska, Chrystopher L. Nehaniv

**Affiliations:** 1 Department of Mechanical and Mechatronics Engineering, University of Waterloo, Waterloo, ON N2L 3G1, Canada; nkliska@uwaterloo.ca; 2 Departments of Systems Design Engineering and of Electrical & Computer Engineering, Waterloo Institute for Complexity and Innovation, University of Waterloo, Waterloo, ON N2L 3G1, Canada

**Keywords:** metabolic boundary, autopoiesis without spatial boundaries, characterization of living systems, self-production, autocatalysis, computational autopoiesis, metabolism, complex systems, emergence of individuality, network science

## Abstract

Ever since Varela and Maturana proposed the concept of autopoiesis as the minimal requirement for life, there has been a focus on cellular systems that erect topological boundaries to separate themselves from their surrounding environment. Here, we reconsider whether the existence of such a spatial boundary is strictly necessary for self-producing entities. This work presents a novel computational model of a minimal autopoietic system inspired by dendrites and molecular dynamic simulations in three-dimensional space. A series of simulation experiments where the metabolic pathways of a particular autocatalytic set are successively inhibited until autocatalytic entities that could be considered autopoietic are produced. These entities maintain their distinctness in an environment containing multiple identical instances of the entities without the existence of a topological boundary. This gives rise to the concept of a metabolic boundary which manifests as emergent self-selection criteria for the processes of self-production without any need for unique identifiers. However, the adoption of such a boundary comes at a cost, as these autopoietic entities are less suited to their simulated environment than their autocatalytic counterparts. Finally, this work showcases a generalized metabolism-centered approach to the study of autopoiesis that can be applied to both physical and abstract systems alike.

## 1. Introduction

In 1971, Kauffman et al. introduced the concept of autocatalytic sets in an effort to explain the origin of life. Autocatalytic sets are defined as networks of chemical species (in the context of biochemistry, microbiology, or abstract chemistry) where members of the set mutually catalyze one another such that the overall set can reproduce itself [1,2].

Alternatively in 1974, Varela et al. introduced the concept of autopoiesis with their computational model of an entity comprising a self-repairing cellular boundary enclosing a dynamic organization that produces and maintains the entity [3,4,5]. Since then, many groups have improved upon their computational original model [6,7,8,9,10,11,12,13,14,15] as well as developed unique models of their own [16]. Autopoiesis builds on autocatalysis with the additional requirement that autopoietic entities can maintain their individuality and identity [17]. Based on this body of work, a definition has emerged, as shown below.

“An autopoietic system is a network of processes which produces all the components whose internal production is necessary to maintain the network operating as a unit.” [18]

Since its inception, there was an assertion that for a system to be considered autopoietic, it must define a boundary that separates itself from its environment [18,19,20,21,22,23,24,25,26]. Even the simplified definition above implies the existence of a boundary by suggesting that an autopoietic entity must have an interior.

In this work, we examine the differences between these two conceptual frameworks and so seek to challenge Varela et al.’s initial framing and assertion that autopoietic systems necessarily require a topological boundary through counterexample. To test our hypothesis, we constructed a novel minimal system, implemented in silico and inspired by chemical and physical interactions at the molecular scale.

One of the core criticisms of autocatalytic systems is that autocatalytic systems typically rely on some external co-location mechanism to ensure that reagents are positioned with respect to one another to efficiently react [2]. As an alternative to cellular co-location (the co-location of chemical species by encapsulation), our model system relies on polymerization to co-locate chemical species relative to one another. Consequently, the resultant entity will feature local dendritic symmetry with chains of particles emanating from central nodes. Moreover, since each particle is in direct contact with the external environment, there is no internal volume and therefore no topological boundary.

**Figure 1:**
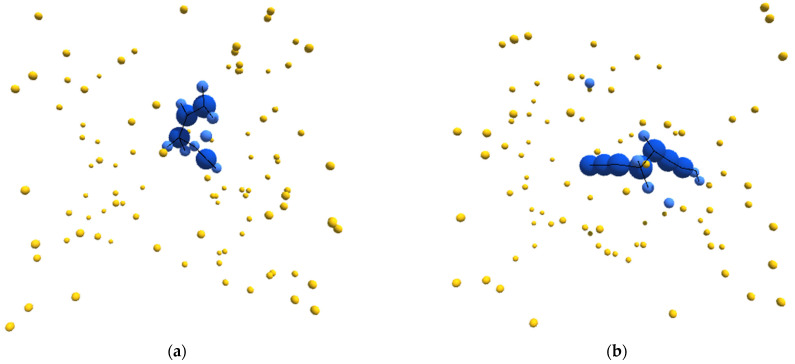

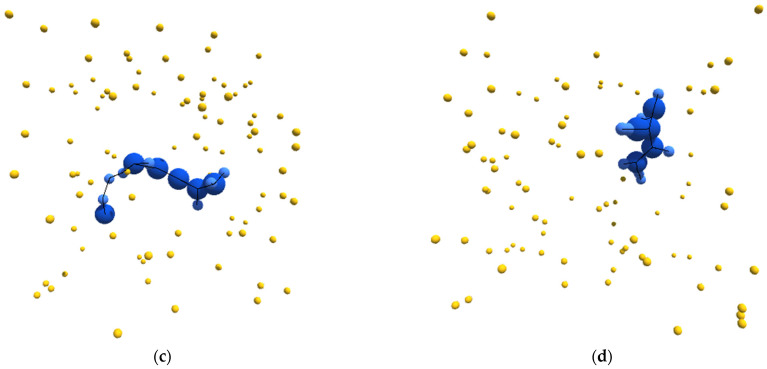
(**a**–**d**) Sample images, from randomly initialized simulations, depicting autopoietic instances derived from the schema used in experiment II. Environmental α-particles are depicted in yellow, β-particles belonging to the depicted instance are shown in light blue, γ-particles belonging to the depicted instance are shown in dark blue, and chemical bonds between neighboring particles are depicted as black lines.

With this as a general framework, we tuned the metabolic pathways of our simulated entities to bring about specific emergent behavior and organization. In particular, we started from a metabolic schema that would unambiguously produce autocatalytic entities. We then blocked certain metabolic pathways until this new schema produced entities that could be considered autopoietic. Figure 1a–d depicts sample structures derived from this schema.

Note, we are using the term schema for the specification of a collection of components (e.g., chemical species) and a collection of transformations (e.g., chemical reaction mechanisms) that describe the metabolism of an entity. An instance of a schema will be a particular realization of that schema in time and space. To aid the reader and maintain precision, a number of such terms are collected in a glossary in Appendix A.

## 2. Experimental Paradigm

To evaluate whether a particular autocatalytic schema belongs to the subset of autopoietic schemata, the heuristic test proposed by McMullin is applied to three related candidate schemata: In each case, two identical instances of the candidate schema will be initialized within the same environment and simulated over a period time where they will be allowed to interact with one another. If the instances can interact without either instance meaningfully losing their individuality (i.e., both instances can be considered internally self-producing), the instances can be said to belong to an autopoietic schema; otherwise, they are merely autocatalytic [17]. Note, two identical instances represent a worst-case scenario where there is no information available with which either external observers or the simulated entities themselves can distinguish themselves and their constituent components from one another. Therefore, each component of an instance will be assigned a ‘color’ so we, as observers, can discern the providence of individual components. These ‘colors’, however, have no effect on the dynamics and only serve to aid in analysis by the experimenters. 

In addition to the colored components that make up each instance, the environment also contains uncolored particles which are the raw materials that the instances consume to produce themselves. These particles are not considered to be a part of any instance.

## 3. Materials and Methods

The model defined subsequently was simulated using a 3D, off-lattice approach reminiscent of molecular dynamic simulations (MD) and was implemented within the Unity engine (version 2020.2.6f1). Unity was chosen as a platform to make use of the native physics engine as well as its built-in visualization capabilities. 

The native physics engine was extended to simulate multibody interactions between rigid body particles in real-time (referred to as the physics layer hereafter). A chemistry layer was implemented on top of this to direct these interactions and handle the transformation of chemical species.

### 3.1. Environment

The environment that instances of the candidate schema inhabit consists of a sealed container (such that no particles can enter or exit the environment) filled with a fluid approximated as an ether that can apply drag forces on all particles within.

In a traditional MD simulation, environmental particles would be simulated and would constrain the motion of the entity particles through collisions. However, due to computing constraints, these other particles were approximated by this ether, and only particles relevant to the candidate schema were simulated.

### 3.2. Particles

Particles are spherical in shape and have a finite collision radius ($r_{C}$), a mass (m), and a linear drag coefficient (D). Furthermore, each particle can move freely in 3-dimensional space but may be subject to multibody interaction forces (bonds) with nearby particles that can act to constrain the position of particles relative to one another.

For two particles to interact with one another, they must be within the sum of their interaction radii ($r_{I}$). Similar to traditional MD simulations, this condition is facilitated through the implementation of a neighbors list. In this study, two separate neighbor lists track both the bound and unbound neighbors of every particle.

Three types of particles were simulated as a part of this study. α-particles are a stable “food” particle native to the environment. On the other hand, instances of the candidate schema comprise a combination of β-particles and γ-particles. β-particles may form up to 2 bonds, while γ-particles can form up to 4 bonds. Table 1 describes the particle parameters used in this study.

### 3.3. Instances

The instances of the candidate schemata modeled in this study are composed of β-particles and γ-particles. Given the physical and chemical interactions that are defined subsequently, these particles will aggregate, through the formation of bonds, into web/dendrite-like structures. While the locality of particles within a given structure is important in order to maintain a self-sustaining structure, these structures may interpenetrate each other; hence, this does not constitute a topological boundary.

Since there are no explicit boundaries in the simulation, individual instances are defined based on their bondedness as only particles connected by chemical bonds can exert forces on each other (as opposed to unbound particles which are considered unconnected). As a result, an individual instance is defined as a set of particles that are mutually bound together and unbound from all other particles in the simulation environment (henceforth referred to as our internality metric). 

These isolated individual instances can be identified and quantified, through graph traversal Algorithm 1 where each particle is treated as a node, and the bonds that connect particles are treated as edges. This algorithm produces a list of isolated connected subgraphs, where each subgraph can be considered to be an individual instance of the candidate schema. Note that this internality metric is only valid for the timestep in which it is calculated and is subject to change over time.

However, to achieve closure under entailment [1,2,27], an individual instance must contain at least one γ-particle. Therefore, only subgraphs that meet this condition will be considered individual instances of the candidate schema (when discussing individual instances hereafter, this condition is implied). Subgraphs that do not meet this condition are not considered viable autocatalytic instances as they cannot continue to produce their own components.
**Algorithm 1.** Organizes collections of particles into isolated subgraphs1**Procedure** Main (list of particles *x*)    **Create**$\;\mathrm{empty}\;\mathrm{list}\;\tilde{x}$    **Create** empty list *y*    **While**
*x* is not empty5        **Create** new empty list *z*        **Select** first element of *x* as *x_i_*        **Append**
*x_i_* to *z*        **Move**
$x_{i}\;\mathrm{from}\;x\;\mathrm{to}\;\tilde{x}$        **Call** Recursive Search (*x_i_*, *z*)10        **Append**
*z* to *y*    **End of Loop**    **Output**
*y***End of Procedure**15**Procedure** Recursive Search (*x_i_*, *z*)    **For Each** particle *z_j_* in *x_i_*. *Bound Neighbors*        **If**$\;\tilde{x}$ does not contain *z_j_*
**Then**            **Append**
*z_j_* to z            **Move**
*z_j_* from x to $\tilde{x}$20            **Call** Recursive Search (*z_j_*, *z*)        **End of If**    **End of Loop****End of Procedure**

### 3.4. Physics Layer

The Unity engine models time as a discrete series of timesteps (50 timesteps per second in our simulation) where all the forces are calculated for each particle, and the positions of all particles are updated simultaneously prior to the next timestep. Note, $\vec{*}$ denotes a vector, while $\hat{*}$ denotes a unit vector.

(1) $${\vec{F}}_{Net}(t)=\left(\sum_{i}^{R,L,T}{\vec{F}}_{i}\left(t\right)\right)-mD\vec{v}(t)$$

In addition to collisions which are handled by the native Unity physics engine, there are three types of physics interaction forces modeled in this study. Equation (1) describes the net force (${\vec{F}}_{Net}(t)$) at timestep t where $\vec{v}(t)$ is the velocity of a particle at timestep t.

#### 3.4.1. Random Walk


(2) $${\vec{F}}_{R}\left(t\right)=c{\vec{F}}_{R}\left(t-1\right)+\left(1-c\right)\vec{w}(t)$$

Diffusion acts as the primary mechanism to counteract the co-location processes presented in this study. To approximate Brownian motion within the ether material, a random force is added to each particle at every timestep to counteract the effect of the drag of the ether material and produce a randomized trajectory. This force (${\vec{F}}_{R}\left(t\right)$) is modeled by a random process described by Equation (2) where c is a constant that describes the temporal correlation of adjacent timesteps, $\vec{w}(t)$ is a random Gaussian vector with $\vec{0}$ mean and covariance matrix $W=wI_{3}$, and w is a rate constant that determines the average speed of the particles. Parameter values c=0.5 and w=25 were modeled in this study.

#### 3.4.2. Linear Spring


(3) $${\vec{F}}_{L\left(AB\right)}=K_{L}\left({\vec{s}}_{AB}-\left(r_{c(A)}+r_{C(B)}\right){\hat{s}}_{AB}\right)$$

(4) $${\vec{s}}_{AB}={\vec{x}}_{B}-{\vec{x}}_{A}$$

**Figure 2:**
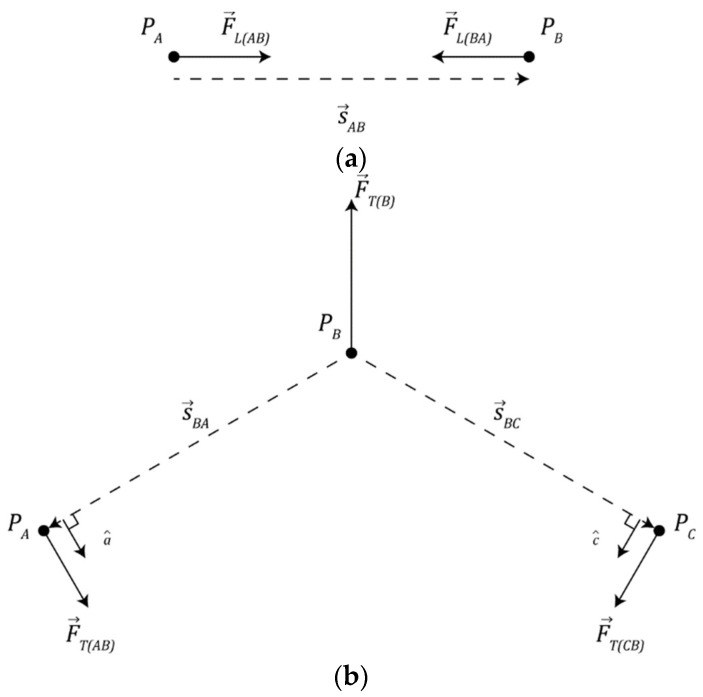
Physical interaction mechanisms involved in chemical bonding: (**a**) diagram of linear spring mechanism and (**b**) diagram of torsion spring mechanism.

Two nearby particles may form a bond, modeled as an ideal linear spring, where they will seek to maintain a fixed distance between one another as depicted in Figure 2a. Equation (3) describes the force exerted on particle A by particle B (${\vec{F}}_{L\left(AB\right)}$) where $K_{L}$ ($K_{L}=100$ was used in this study) is the linear spring constant of the bond, ${\vec{s}}_{AB}$ is the vector that points from particle A to particle B, as in Equation (4), and ${\vec{x}}_{A}$ and ${\vec{x}}_{B}$ denote the positions of particles A and B, respectively. Note, to improve computational efficiency, the simulation makes use of Newton’s second law where we note ${\vec{F}}_{L\left(BA\right)}=-{\vec{F}}_{L\left(AB\right)}$.

#### 3.4.3. Torsional Spring

Once a particle has bonded to 2 or more other particles, the placement of bonds becomes important. A three-body force, modeled as a torsion spring, ensures that the angular distance between bonds is maximized. This force gives collections of connected particles a characteristic shape akin to the carbon spine of organic molecules in the real world.

Figure 2b depicts a collection of particles *A*, *B*, and *C* where particles *A* and *C* are bonded to particle *B*, the torque (τ) produced by the torsion spring is given by Equation (5) where $K_{T}$ ($K_{T}=3$ was used in this study) is the spring constant of the torsion spring, ${∠}_{ABC}$ describes the angle subtended by particles *A*, *B*, and *C*, and ${∠}_{B}^{*}$ denotes a target angle dependent on the number of particles bound to particle B (*N_B_*).

(5) $$\tau =K_{T}\left({∠}_{ABC}-{∠}_{B}^{*}\left(N_{B}\right)\right)$$

(6) $${\vec{F}}_{T\left(AB\right)}=\frac{\tau }{\left\|{\vec{s}}_{BA}\right\|}\hat{a}$$

(7) $${\vec{F}}_{T\left(CB\right)}=\frac{\tau }{\left\|{\vec{s}}_{BC}\right\|}\hat{c}$$

(8) $${\vec{F}}_{T\left(B\right)}=-{\vec{F}}_{T\left(AB\right)}-{\vec{F}}_{T\left(CB\right)}$$

This torque exerts a force on each particle in the collection. Equation (6) describes the force (${\vec{F}}_{T\left(AB\right)}$) exerted on particle *A* by particle *B*, Equation (7) describes the force (${\vec{F}}_{T\left(CB\right)}$) exerted on particle *C* by particle *B*, and Equation (8) describes the recoil force (${\vec{F}}_{T\left(B\right)}$) on particle *B* that counterbalances the other forces produced by the torsion spring.

The values of ${∠}_{B}^{*}$ were chosen so that when there are 2, 3, and 4 neighbors, a linear, triangular, and tetrahedral configuration will be produced, as shown in Table 2.

### 3.5. Chemistry Layer

**Figure 3:**
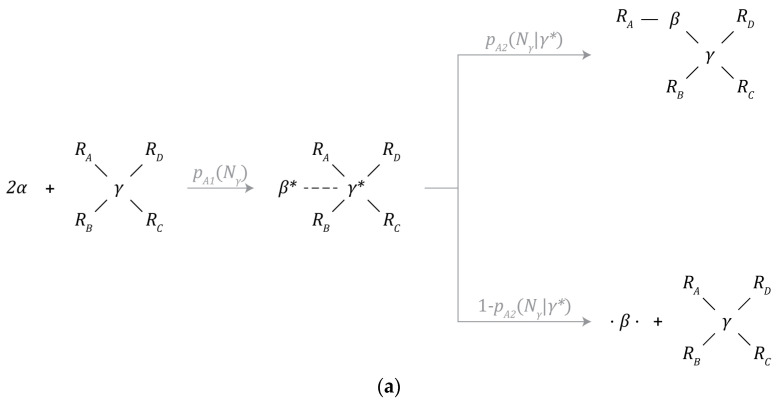

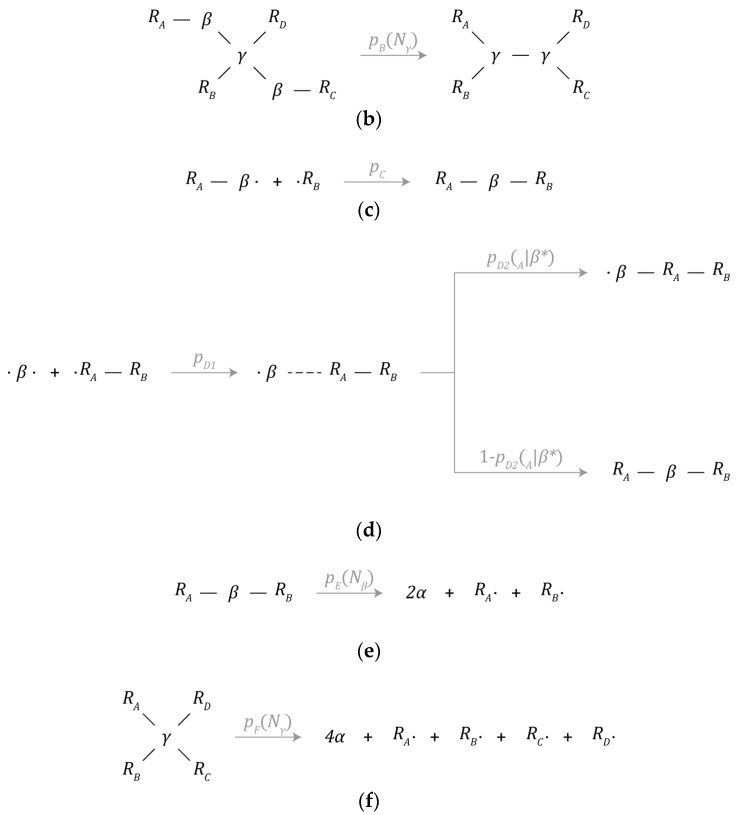
Chemical reaction mechanisms: (**a**) β-particle synthesis, (**b**) γ-particle synthesis, (**c**) partially bonded β-particle bonding, (**d**) free β-particle bonding, (**e**) β-particle decay, and (**f**) γ-particle decay.

Figure 3a–f depict the set of chemical reaction mechanisms that can create or destroy particles and chemical bonds and transform one type of particle to another within the simulated environment. All chemical reactions take one timestep to complete, are calculated prior to the physics update, and each particle can only initiate one chemical reaction mechanism each timestep. Since a series of reactions can lead to drastically different results depending on the order of operations, this restriction greatly simplifies the implementation of the model.

The chemistry layer is primarily implemented within the bound and unbound neighbor lists of each particle. As a result, each particle can be treated as a Markov decision process where the behavior arbitration policy is set by the probability of a chemical reaction being initiated, while the probability of any given outcome is determined by the physics layer, as depicted in Figure 3a,b. Note, behavior arbitration is not influenced by the unbound neighbor’s list and is purely random.

Finally, given that particles need to be within each other’s interaction volumes to interact, if two bound particles leave each other’s interaction volumes, then the bond between them will also be severed.

#### 3.5.1. β-Particle Synthesis

A β-particle is created through the combination of 2 α-particles. This process is catalyzed by a γ-particle, as depicted by Figure 3a. Under this mechanism, α-particles are first incorporated into the structure of the γ-particle where one is destroyed while the other is transformed into a β-particle. The probability of this mechanism occurring given that there are at least 2 α-particles within the interaction volume of the γ-particle is denoted by $p_{A1}$ and is a function of the number of neighbors ($N_{\gamma }$) bound to the γ-particle.

At this metastable intermediate point, the newly created β-particle can either be incorporated into the structure of the instance containing the γ-particle that catalyzed its formation, or the β-particle can be ejected into the environment. The probability of incorporation is denoted by $p_{A2}$ and is a function of the number of neighbors ($N_{\gamma }$) bound to the γ-particle. This incorporation event can only take place during the synthesis of the β-particle. The probability of ejection is the complement of the probability of incorporation.

Table 3 summarizes the values of $p_{A1}$ and $p_{A2}$ used in this study. $p_{A1}$ was tuned to bias the expansion of instances by favoring the availability of β-particles around newer and less established γ-particles. $p_{A2}=0.25$ was used in this study to showcase the possibility of β-particles produced by one instance to be incorporated into another.

#### 3.5.2. γ-Particle Synthesis

A γ-particle can split into 2 new, bound γ-particles by consuming 2 adjacently bound β-particles, as depicted in Figure 3b. The probability of this process occurring is given by $p_{B}$ and is a function of the number of neighbors ($N_{\gamma }$) bound to the γ-particle.

Table 4 summarizes the values of $p_{B}$ used in this study and was tuned to optimize the viability of newly formed γ-particles.

#### 3.5.3. Bond Formation

Aside from bond formation occurring as a by-product of the mechanisms discussed above, bond formation can also be initiated by β-particles in one of two ways.

The first mechanism is depicted in Figure 3c (partially bonded β-particle bonding) and will be referred to as addition hereafter. A β-particle with one available bonding site can initiate a bond with a neighbor particle (particle $R_{B}$) with the interaction volume of the β-particle. This other particle must also have at least one available bonding site for bond formation to occur. The probability of bond formation given all the conditions described above is given by $p_{C}$.

The second mechanism is depicted in Figure 3d (free β-particle bonding). A β-particle with no bound neighbors can interact with a neighbor particle (particle $R_{A}$) with the interaction volume of the β-particle. One of two things can happen; bond formation can proceed just as described above where the β-particle establishes a new bond at an available bond site around particle $R_{A}$. Alternatively, the β-particle can insert itself between particle R_A and its neighbor nearest to the β-particle. The probability of initiation is given by $p_{D1}$, while the probability of addition is given by $p_{D2}$ and is dependent on the number of bound neighbors ($N_{A}$) surrounding particle $R_{A}$. The probability of insertion is the complement of the probability of addition.

Table 5 summarizes the values of $p_{C}$ and $p_{D1}$ used in this study, where these parameters were chosen to quantify the chemical potential of β-particles. As such, it is expected that β-particles with no bound neighbors would be more volatile and exhibit a greater propensity for bonding than β-particles that already have bound neighbors.

Table 6 summarizes the values of $p_{D2}$ in this study and varies depending on whether the target particle is a β-particle or a γ-particle and was tuned to optimized network diversification.

Mechanism C (partially bonded β-particle bonding) is a metabolic pathway that gives instances the ability to repair themselves from damage through the reattachment of previously severed parts. However, this can also lead to the fusion of unrelated instances due to proximity. Similarly, mechanism D (free β-particle bonding) is a metabolic pathway that gives instances the ability to capture useful intermediate products from the environment rather than manufacturing them internally. As such, the providence of these intermediate products is not restricted to that of the instance that produced them.

#### 3.5.4. Particle Decay

β-particles and γ-particles can spontaneously decay back into 2 or 4 α-particles, respectively, as depicted in Figure 3e,f. Upon decaying, bonds with adjacent neighbors will be severed. These mechanisms represent the primary means in which simulated instances of the candidate schema may be damaged or destroyed.

The probability of β-particle decay is denoted by p_E and is a function of the number of bound neighbors ($N_{\beta }$) around a particular β-particle. Similarly, the probability of γ-particle decay is denoted by p_F and is a function of the number of bound neighbors ($N_{\gamma }$) around a particular γ-particle. These parameters were tuned to be proportional to chemical volatility. Note, the choice of $p_{E}$ and $p_{F}$, described in Table 7, rewards the establishment of new bonds, and as a result, the formation of strongly interconnected instances is universally optimal under these conditions. In conjunction with the parameter values described in mechanisms C and D, instances are likely to expand and are encouraged to grow, potentially indefinitely, or to some equilibrium value as the limited amount of available material allows for.

### 3.6. Particle Color

To assess the providence of β-particles and γ-particles produced throughout the simulation, a color property will be introduced. Particles can either be red (color value 1) or blue (color value −1). The color of a particle is determined by the color of the parent particle at the point of creation. The color of β-particles is determined by the color of the γ-particle that catalyzed its creation. Similarly, the color of γ-particles will be determined by the color of the γ-particle from which they split.

Note, the color property is purely for record keeping and is not used as a means to discriminate potential chemical interactions.

### 3.7. Outputs and Post-Processing

The simulation produced a real-time animation of instance(s) derived from each of the 3 candidate schemata. Additionally, simulation data (including the particle species, color, position, and list of bound neighbors of each particle) were post-processed to identify individual instances and characterize their instantaneous properties at each sampled timestep. Additionally, to quantify the competency of the instances within the simulation, the mass of all instances was calculated as well.

Furthermore, to examine the purity of the color value within the simulation, each individual instance was binned into one of two groups: majority red or majority blue corresponding to the color values of the initial seed γ-particles. An instance is majority red if it comprises a greater number of red γ-particles than blue γ-particles. Conversely, an instance is considered majority blue if it comprises a greater number of blue γ-particles than red γ-particles. If an instance comprises an equal number of red γ-particles and blue γ-particles, then it is neither considered majority red or majority blue and is not considered in our analysis.

(9) $$N(x)=\mathrm{count}\left(G\in x\right)$$

(10) $$S_{i}=\mathrm{count}\left(P\in G_{i}\right)$$

(11) $$M(y)=\sum m(P_{j})\;\forall P\in y$$

(12) $$C_{i}(x,y)=\left\{\begin{array}{cc} \frac{\sum C(P_{j})\forall P\in G_{i}\cap y}{\mathrm{count}\left(P\in G_{i}\cap y\right)} & if\;G_{i} \end{array}\right.\in x$$

Equation (9) describes the number of instances of color *x* (N(x)) where x is the majority color of the individual instance (either red, blue, or all), and G denotes the set of individual instances present at the sampled frame (as determined by Algorithm 1). Equation (10) describes the size ($S_{i}$) of individual instance i ($G_{i}$) where P is the set of particles present in the simulation. Equation (11) describes the total mass (M(y)) of particle species y (β-particles or γ-particles), where $m\left(P_{j}\right)$ is the mass of the $j^{th}$ particle. Equation (12) describes the average color value ($C_{i}(x,y)$) of particle species y within an individual instance i which is majority color x where $C(P_{j})$ denotes the color value of the $j^{th}$ particle.

## 4. Results

### 4.1. Initial Conditions

The experimental results described hereafter are all based on simulations stemming from the same set of initial conditions containing 1000 α-particles, 1 red γ-particle, and 1 blue γ-particle all within a cubic container of side length 40 length units. Each set of results is based on a sample simulation in which both red and blue particles survived for the duration of the simulation.

### 4.2. Experiment I

The instances in experiment I implement the base autocatalytic schema with the reaction mechanism parameters described in Section 3.5 (the schema uses the parameters from the first row of Table 5). As such, these instances allow for reaction mechanisms C and D (partially bonded β-particle bonding and free β-particle bonding, respectively).

**Figure 4:**
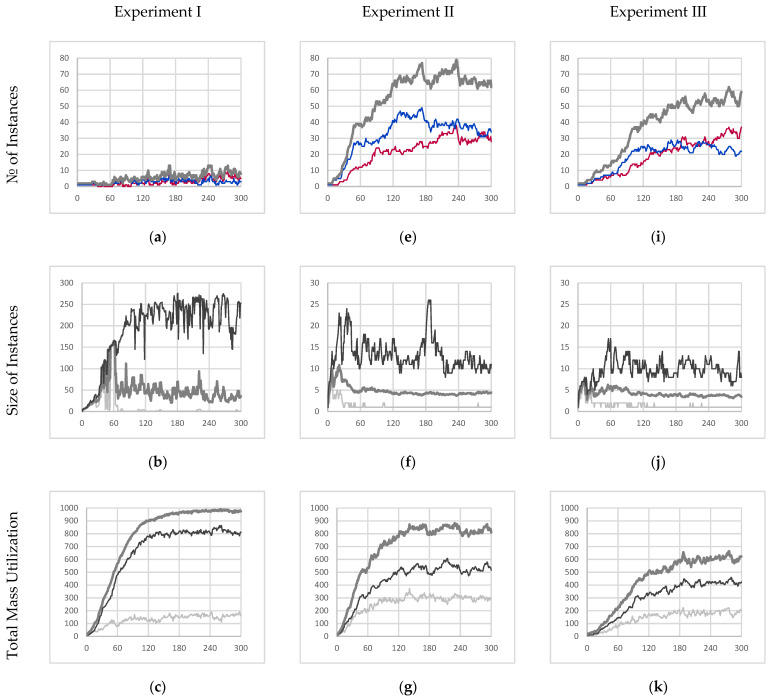

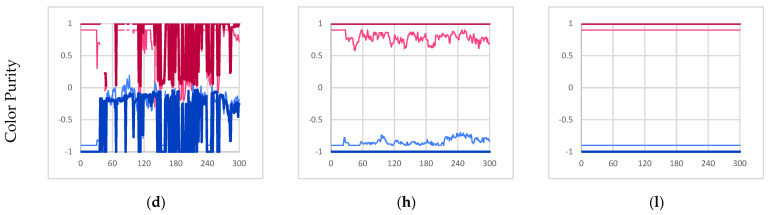
Experimental results for experiments I, II, and III. (**a**,**e**,**i**) The time evolution of the quantity of individual instances [majority red instances in red, majority blue instances in blue, all individual instances in thick medium gray]. (**b**,**f**,**j**) The time evolution of the size of individual instances [maximum in black, average in medium gray, minimum in light gray]. (**c**,**g**,**k**) The total mass utilization out of 1008 potential available mass units [total mass of β-particles in light gray, total mass of γ-particles in black, all particles in thick medium gray]. (**d**,**h**,**l**) The time evolution of the average color value within majority red and majority blue individual instances [majority red average γ-particle color value in dark red, majority red average β-particle color value in light red, majority blue average γ-particle color value in dark blue, majority blue average β-particle color value in light blue]. Note, β-particle color values range from −0.9 to 0.9 for visual clarity. All figures depict 300 s of simulation data sampled once every second.

As expected, this resulted in a collective instance deriving from an autocatalytic schema (since the collective instance fails to meet the condition of internal production) and whose performance is summarized by Figure 4a–d. Figure 4a,b describe an emergent behavior where the collective instance cyclically fractures and recombines, which suggests that any notion of individuality would not apply meaningfully. Moreover, Figure 4b shows that most of the particles available in the simulation are part of the core entity. During the final minute of simulation, it was found that the core entity comprised 226.85 particles on average with a standard deviation of 34.84 particles.

As a result of this cycle of fracture and recombination, the entity has no way to prevent itself from fusing with other nearby instances. In the experiment, it was found that the red and blue instances merged and became one collective instance due to their proximity. This is best described by Figure 4d which shows that the average color value of the collective instance fluctuates wildly (no further quantitative analysis was conducted on the color purity data). However, this result clearly shows that individuality was not maintained.

While not autopoietic, we can see that the instance is very well adapted to survive in the environment. Figure 4c shows that the collective instance eventually consumed roughly 97.1% percent of all resources available within the sealed simulation environment. During the last minute of simulation, the collective entity had an average mass of 978.69 mass units with a standard deviation of 6.33 mass units. Not only does this represent the highest total mass (as compared to other experiments) but also the lowest amount of volatility. This can be attributed to the inclusion of reaction mechanism C (partially bonded β-particle bonding) which allows for fusion with nearby instances, the healing of ruptured particle chain segments, and the formation of loop structures within an instance that all serve to minimize the decay rate of particles in the network. As a result of this decrease in overall decay rate, 83.8% of the mass of the collective entity is made up of γ-particles (16.2% β-particles).

### 4.3. Experiment II

The instances in experiment II implement a modification of the base schema, as described in Section 3.5, where mechanism C (partially bonded β-particle bonding) is suppressed (this new schema uses the parameters from the second row of Table 5).

The results of this experiment are described in Figure 4e–h. Experiment II represents an intermediate step in between a collective instance that derives from an autocatalytic schema and a series of individual autopoietic instances that are purely self-sustaining.

The inhibition of mechanism C prevents the fusion of any instance (bonded network of particles) regardless of color. As a result, this prevents the mixing of red and blue γ-particles within the same instance, as shown in Figure 4h. However, the inclusion of mechanism D (free β-particle bonding) still allows for free β-particles to insert themselves into unrelated instances (instances that did not produce those particular β-particles). Consequently, we still observe red and blue β-particles within the same instance. Note, however, since the color of newly formed γ-particles is not dependent on the β-particles consumed in the reaction, the color purity of γ-particles within a given instance is guaranteed. For example, in a majority blue individual instance, blue γ-particles can consume incorporated foreign red β-particles to recover the overall color purity of the instance over time.

Without reaction mechanism C, severed instances may continue to exist as separate individual instances (provided they both contain at least one γ-particle each). This manifests itself as an emergent mechanism of reproduction through fragmentation. Figure 4e shows how the number of individual instances increases exponentially before reaching an average carrying capacity of the environment of 65.07 individual instances with a standard deviation of 1.81 instances.

Consequently, these newly formed instances will be much smaller (as compared to the collective instance from experiment I), as depicted in Figure 4f, where the average steady state instance size is only 4.29 particles, and even the largest instances only contained 11.13 particles on average.

Lastly, Figure 4g depicts that the schema from which the instances in the experiment are derived is not as well suited to the environment. The sum of all individual instances only accounts for 81% of the total mass within the environment on average at steady state (mean of 818.30 and standard deviation of 21.25 mass units at steady state) and is significantly more volatile. Consequently, it was observed that this increased volatility resulted in only 63.7% (by mass) of all the individual instances comprising γ-particles on average at steady state (36.3% β-particles). With the reduced interconnectivity within instances, particles are more likely to decay and as a result less likely to be eventually promoted to γ-particles.

### 4.4. Experiment III

The instances in experiment III implement a further modification of the base schema, as described in Section 3.5, where mechanisms C and D (partially bonded β-particle bonding and free β-particle bonding, respectively) are suppressed (this new schema uses the parameters from the third row of Table 5). The results of this final experiment are depicted in Figure 4i–l.

Like experiment II, the exclusion of mechanism C (partially bonded β-particle bonding) prevents the fusion of disparate instances thus preventing the mixing of red and blue γ-particles within a given instance. Furthermore, the additional inhibition of mechanism D (free β-particle bonding) prevents free β-particles from being incorporated into any instance. As a result, the only available metabolic pathway for β-particles to be incorporated into a given instance is through mechanism A (β-particle synthesis). Consequently, mechanism A only incorporates β-particles produced by γ-particles that are already members of a given instance; therefore, the resulting instances are internally producing. As expected, Figure 4l shows that all instances maintain the purity of color throughout the entire simulation further demonstrating internal production. Given that the instances meet the internal production criterion, and they are already self-co-locating autocatalytic instances, we can conclude that they derive from an autopoietic schema.

This comes at a cost, however, where the absorption efficiency of those produced β-particles is severely decreased. Like the previous experiment, Figure 4i depicts the evolution of the population of instances throughout the simulation with an average carrying capacity of 54.43 instances with a standard deviation of 3.14 instances. This shows that the schema from which these entities derive is even less well suited to the environment. The instances in this experiment are even more likely to “die” and be recycled than in the previous experiment.

Furthermore, those unabsorbed β-particles sequester away α-particles preventing further attempts at absorption until the β-particles decay in time. This amounts to an increased scarcity of food particles available at any given time resulting in even smaller instance sizes, as depicted in Figure 4j, where the average instance size is only 3.69 particles, and even the largest entities are only 9.30 particles on average.

Not surprisingly, the collective mass utilization of all instances in this simulation was only 61% of the available mass within the simulation (mean of 611.11 with a standard deviation of 19.64 mass units) at steady state. This is summarized in Figure 4k. Lastly, it is observed like in the previous experiment, 68.9% of each instance (by mass) comprised γ-particles (31.1% β-particles) suggesting that the overall structure of instances was similar to those in experiment II, suggesting that the inhibition of mechanism D did not affect the organization of instances but only their overall competency with their environment.

## 5. Discussion

Firstly, it is important to note that the implementation of the chemistry layer resembles an agent-based architecture, where each particle is treated as a software agent, the unbound neighbor’s list acts as a sort of a sensorium, the bound neighbor’s list acts as sort of internal memory, and the chemical reaction mechanisms available to each particle act as a sort of motorium for each software agent. This approach minimizes the computational complexity of the overall simulation and is computationally equivalent to a loop that updates each particle present in the simulation. Such implementations are typical in molecular dynamic simulations.

A fundamental limitation of the simulation is that given the reaction mechanisms present in the study, there are not enough degrees of freedom to limit the growth of a collective instance (such as the one from experiment I) without making the environment unhabitable. As a result, this will produce a single instance that consists of the vast majority of the mass of the collective instance. Even under the right conditions, a self-co-locating autocatalytic set could be designed to exist as a collection of smaller instances; however, the membership of those instances would be in constant flux, and consequently, there is no meaningful boundary.

It is important to address that experiment I featured the most well-adapted entity for the given environment (note that the simulated environment does not contain any particles/structures that are harmful to the simulated instances). Since all variations of the base schema in this study share the same localization method and given reaction mechanisms E and F (β-particle decay and γ-particle decay, respectively), it is universally true that a larger instance is likely to be more interconnected, therefore more stable, and consequently better able to monopolize resources. While bigger may not be better in all situations, self-localizing autocatalytic schemata are able to take advantage of more opportunities than autopoietic schemata since they have access to metabolic pathways that would open the instance to merging with nearby entities. The collective instance in experiment I was able to produce itself by scavenging for fully functional parts that it could incorporate into its internal structure. Conversely, the instances in experiment III needed to wait for these parts to be recycled and then rebuilt within their own internal structures. This is less efficient but does guarantee the maintenance of their individuality.

Experiment III did produce instances that could be considered autopoietic. Those instances did exhibit some sort of boundary regions that separated individual instances from the rest of the simulation. Even without any internal volume, each instance features a manifold that contains all its constituent particles within. However, since these particles are directly exposed to the simulation environment, there is no meaningful topological boundary. Instead, the metabolic schema used in experiment III produces an emergent sort of selection criteria for the incorporation of foreign particles into the network of the instance.

Through a combination of local interactions (chemical bonds) and internal production, this selection criteria acts as a self-selection algorithm/mechanism without the need for identifying labels and without a topological boundary. As such, this mechanism is not constrained by the number of unique labels that can be resolved, rather the selectivity of the underlying chemical reaction mechanisms.

The availability of metabolic pathways determines the kinds of materials that may be incorporated into an autocatalytic instance thus achieving distinction from its surrounding environment. Furthermore, as was demonstrated in experiment III, the inhibition of certain metabolic pathways can result in an emergent metabolic boundary capable of differentiating between indistinguishable instances of the same autopoietic schema. As a result, a metabolic boundary represents an alternative solution to the problem of creating the distinction necessary for autopoietic systems.

Interestingly, since the color value of consumed β-particles is subsumed by the active γ-particle in reaction mechanism B, it can be concluded that β-particles do not meaningfully contribute to the color value of a given instance. Furthermore, the results of experiment II and experiment III demonstrate that the organization/structure and of instances is not affected by the inclusion of mechanism D (free β-particle bonding). This suggests that β-particles may also be treated as “food” particles, and their providence is irrelevant. With this modified definition of environmental particles, the schema presented in experiment II would also satisfy the definition for an autopoietic schema.

More broadly, this would suggest that any particle that does not carry any genetic or structural/organizational information can be considered a substrate particle or “food” particle. Moreover, given the disparity in competence between instances produced by experiment II as compared to those of experiment III, it is optimal for an autopoietic schema to minimally discriminate against environmental particles that do not impact the structure/organization of the derived instances to better take advantage of all possible opportunities available within in the environment. As such, it is important to point out that the bias of the observer in defining what constitutes the environment can have a material impact on the results of studies aimed at identifying autopoietic entities.

At the outset of this study, we defined an internality metric for distinguishing a collection of particles from the environment and assigning them as belonging to a particular instance of an autocatalytic schema. We designed our internality metric to identify networks of bound particles due to the unavailability of a topological boundary (in traditional cellular autopoietic systems, an internality metric would select particles encapsulated by the topological boundary of the autopoietic instance of interest). While this metric was the natural choice for our minimal model (as no other interaction forces were modeled aside from chemical bonds), real-world biological systems employ a multitude of phenomena to constrain chemical species. As a result, it is unclear what an appropriate metric would be for such a system. If a metric is too restrictive, it may be impossible to identify autocatalytic instances as they may be too small to achieve closure under entailment. Conversely, if the metric is too permissive, then it may be impossible to define a boundary that separates entities from one another, or from the environment itself, and thus no autopoietic instances could be identified. Alternatively, there may exist multiple valid internal metrics that satisfy both above criteria resulting in multiple ways to classify autopoietic entities within the same system. Therefore, the design of an internality metric is another example of where the bias of the observer may influence the results of a study aimed at identifying autopoietic entities.

The simulation model also suggests that it may be impossible in practice to experimentally classify whether a given instance is purely autopoietic based on McMullin’s heuristic [17] since categorization may require access to some inaccessible internal states of the instance or its constituent components. The results presented here rely on bond information and color values, which would be difficult to access without full knowledge of the simulated universe. Furthermore, autopoiesis was achieved through the complete inhibition of reaction mechanisms C and D (partially bonded β-particle bonding and free β-particle bonding, respectively). However, if their probabilities were not known to the observer, it would be impossible discern a small non-zero probability from complete mechanism inhibition. Therefore, in practice, it is only necessary that a system be substantially autopoietic, meaning that boundaries are only partially effective resulting in a non-zero probability of the recombination of separate autopoietic instances. For example, consider the phenomena of sexual parasitism observed in some species of deep sea ceratioid anglerfish where the males will attach to females resulting in the fusion of epidermal tissues and the eventual uniting of their circulatory systems [28]. Rather than classifying a system as autopoietic or autocatalytic, it may be more appropriate to measure the degree to which a system can be considered autopoietic (or the degree to which a system is able to maintain its individuality).

This study presents an alternative, metabolism-centered framework for examining minimal autocatalytic/autopoietic systems in silico. The keys to this approach are defining a candidate system as a network of reaction mechanisms, coupled with the design of a generalized internality metric tailored to the simulation environment. Together, these tools allowed us to demonstrate the theoretical existence of a class of autopoietic schemata without topological boundaries. This framework allows us to abstract away the construction of a boundary layer and study the metabolic pathways crucial to maintaining this boundary. In the future, our minimal model could be extended to study other properties of minimal autocatalytic/autopoietic systems. Furthermore, our framework itself could be extended to study systems at other scales through the design of new physics and chemistry layers. In this way, the physics and chemistry layers may be coarse-grained to study multicellular organisms. Even more interestingly, abstract rulesets may be substituted in place of the physics and chemistry layers to study simulated autopoietic/autocatalytic systems such as swarms of software agents [29,30] or social systems [31,32].

Finally, it is worth remarking that differentiated multicellular life exists where individuals are structured as networks, such as the mycelium of fungi, which supports the external transformation and digestion of resources for nutrient absorption. This contrasts with a topological boundary enclosing an internal space where food is digested, as is typical of animals. Structures similar to fungal mycelium dating back over two billion years appear to characterize some of the earliest known forms of multicellular life on earth [33], with network structures comprising “jumbles of tangled threads that branch and rejoin”. This suggests there is merit in a further re-examination of the notion of boundaries in individuality and autopoiesis: Structures other than the roughly spherical boundary of a living cell, such as non-simply connected surfaces in three dimensions, as well as branching structures and dynamically changing networks as in fungal mycelium, offer examples of alternative realizations and roles for spatial and metabolic boundaries in autopoiesis that must be considered in fully understanding the possible diversity in the nature of autopoiesis in life on earth and wherever else in the universe it may be found.

## Figures and Tables

**Table 1:** Physical properties of particles.

Property	α-Particles	β-Particles	γ-Particles
$r_{C}$ [length units]	0.25	0.5	1
$r_{I}$ [length units]	1	2	3
m [mass units]	1	2	4
D [1/s]	1	2	4

**Table 2:** Torsion spring target angle as a function of bound neighbors.

*N_B_*	${∠}_{B}^{*}\left(N_{B}\right)$
2	180°
3	120°
4	109.4712°

**Table 3:** The probability of β-particle synthesis initiation as a function of the number of bound neighbors around the initiating γ-particle.

$N_{\gamma }$	$p_{A1}(N_{\gamma })$/Timestep
0	1
1	0.5
2	0.25
3	0.125
4	0.0625

**Table 4:** The probability of γ-particle synthesis initiation as a function of the number of bound neighbors around the initiating γ-particle.

$N_{\gamma }$	$p_{B}(N_{\gamma })$/Timestep
0	0
1	0
2	1 × 10^−3^
3	4 × 10^−3^
4	16 × 10^−3^

**Table 5:** Probability of partially bonded and free β-particle bonding initiation for each experiment.

Experiment	$p_{C}$/Timestep	$p_{D1}$/Timestep
I	4 × 10^−2^	8 × 10^−2^
II	0	8 × 10^−2^
III	0	0

**Table 6:** The probability of addition given free β-particle bonding has been initiated as a function of the number of bound neighbors around the target particle.

$N_{A\beta }$ $/N_{A\gamma }$	$p_{D2}(N_{A\beta })$	$p_{D2}(N_{A\gamma })$
0	1	1
1	0.5	0.875
2	0	0.75
3	--	0.5
4	--	0

**Table 7:** The probability of β-particle and γ-particle decay as a function of the number of bound neighbors around the target particle.

$N_{\beta }$ $/N_{\gamma }$	$p_{E}\left(N_{\beta }\right)$/Timestep	$p_{F}\left(N_{\gamma }\right)$/Timestep
0	16 × 10^−3^	16 × 10^−4^
1	4 × 10^−3^	8 × 10^−4^
2	1 × 10^−3^	4 × 10^−4^
3	-	2 × 10^−4^
4	-	1 × 10^−4^

## Data Availability

The original software and data presented in the study are openly available from https://git.uwaterloo.ca/SIRRL/kliska-nehaniv-autocatalysis-autopoiesis-biosystems, accessed on 22 May 2024.

## Appendix A. Glossary

Since our system has no internal volume, we need to modify the definition of autopoiesis since the existing definition presupposes an internal volume and therefore a topological boundary. Furthermore, in an effort to disambiguate the substance of this report from the myriad of competing definitions in the literature, a glossary of terms is provided for the reader’s convenience.

- **Schema:** A blueprint comprising a collection of components (e.g., chemical species) and a collection of transformations (e.g., chemical reaction mechanisms) arranged into a network that describes the metabolism of an entity.
- **Transformation:** A process that converts one component (or group of components) into another type of component (or group of components) that is enabled by those affected components being in proximity to one another. In addition to component proximity, there may also be some external conditions required for the transformation to proceed.
- **Instance:** An instance of an entity described by a schema is a particular realization of that schema in time and space. In our setting, each constituent component in an instance is chemically bonded to all other constituent components either directly or through a series of intermediary components.
- **Autocatalytic Schema:** A schema where its elements mutually catalyze the synthesis of one another such that the overall set can produce itself (this is also referred to as closure under entailment [1,2,27]).
- **Autopoietic Schema:** A subset of autocatalytic schemata which are also self-co-locating and internally producing.
- **Self-Co-location:** A schema is said to be self-co-locating if its derived instances can constrain their constituent components in proximity to one another. Alternatively, the components themselves may have some property that allows them to constrain other components in proximity to themselves.
- **Internal Production:** A schema is said to be internally producing if its derived instances comprise components produced by the instance which they are a part of. More specifically, each component must be synthesized by another component (or group of components) that is chemically bonded to the relevant instance.
- **Individual Instance:** An instance which remains isolated from other instances over relevant time scales.
- **Collective Instance:** An instance whose membership fluctuates significantly over relevant time scales, where it may continually fragment, recombine, or even incorporate other instances of the same schema that were never previously associated with the original. In such a system, there is no meaningful concept of individuality.
